# UV-C and Nanomaterial-Based Approaches for Sulfite-Free Wine Preservation: Effects on Polyphenol Profile and Microbiological Quality

**DOI:** 10.3390/molecules30020221

**Published:** 2025-01-08

**Authors:** Kamila Pachnowska, Jolanta Kochel-Karakulska, Adrian Augustyniak, Valentina Obradović, Ireneusz Ochmian, Sabina Lachowicz-Wiśniewska, Ireneusz Kapusta, Klaudia Maślana, Ewa Mijowska, Krzysztof Cendrowski

**Affiliations:** 1 Department of Nanomaterials Physicochemistry, Faculty of Chemical Technology and Engineering, West Pomeranian University of Technology in Szczecin, Piastów 45, 70-311 Szczecin, Poland; kamila.pachnowska@zut.edu.pl (K.P.); klaudia.maslana@zut.edu.pl (K.M.); 2 Department of Horticulture, Faculty of Environmental Management and Agriculture, West Pomeranian University of Technology in Szczecin, Juliusza Słowackiego 17, 71-434 Szczecin, Poland; ireneusz.ochmian@zut.edu.pl; 3 Department of Microbiology and Biotechnology, Faculty of Biotechnology and Animal Husbandry, West Pomeranian University of Technology in Szczecin, Piastów 45, 70-311 Szczecin, Poland; 4 Department of Chemical and Process Engineering, Faculty of Chemical Technology and Engineering, West Pomeranian University of Technology in Szczecin, Piastów 42, 71-065 Szczecin, Poland; adrian.augustyniak@zut.edu.pl; 5 Center for Advanced Materials and Manufacturing Process Engineering (CAMMPE), Piastow Avenue 42, 71-065 Szczecin, Poland; 6 Faculty of Tourism and Rural Development in Požega, Josip Juraj Strossmayer University of Osijek, Vukovarska 17, 34 000 Požega, Croatia; 7 Department of Medicine and Health Science, Calisia University (University of Kalisz), Nowy Świat 4, 62-800 Kalisz, Poland; s.lachowicz-wisniewska@uniwersytetkaliski.edu.pl; 8 Department of Biotechnology and Food Analysis, University of Economy and Business, Komandorska 118/120, 53-345 Wrocław, Poland; 9 Department of Food Technology and Human Nutrition, College of Natural Science, Rzeszow University, Zelwerowicza 4, 35-601 Rzeszow, Poland; ikapusta@ur.edu.pl; 10 Faculty of Civil and Environmental Engineering, West Pomeranian University of Technology in Szczecin, Piastów 50a, 70-311 Szczecin, Poland; krzysztof.cendrowski@zut.edu.pl

**Keywords:** wine, *Saccharomyces cerevisiae*, photocatalysis, food preservation, polyphenols, bioprocess engineering

## Abstract

Controlling the microorganisms employed in vinification is a critical factor for successful wine production. Novel methods aimed at lowering sulfites used for wine stabilization are sought. UV-C irradiation has been proposed as an alternative for reducing the viable cell count of microorganisms in wine and grape juice. Nevertheless, UV-C treatment poses the risk of altering the chemical properties of wine. Therefore, this study aimed to test and implement iron oxide–silica core–shell nanomaterial functionalized with TiO_2_ in UV-C treatment of white and red wines. Material for the study consisted of the synthesized nanocomposite, *Saccharomyces cerevisiae* as a model yeast, and Muscaris and Cabernet Cortis wines. The viability of yeasts under treatment, the physiochemical properties of wine, and polyphenol content were tested. Studies have shown that nanomaterial can modulate the effects of UV-C treatment regarding yeast viability and polyphenol content, and the effectiveness of the treatment depends on the wine type. These results open up discussion on the possible use of the proposed hurdle technology in winemaking to control the polyphenol composition and alcohol reduction.

## 1. Introduction

Wine production is based on alcoholic fermentation, a microbiologically complex process which takes place with the participation of yeast and requires the monitoring of microorganisms at various stages [1,2,3,4,5]. One of the most commonly used microorganisms in grape fermentation processes is *Saccharomyces cerevisiae* [2,3]. In most cases, clarification and stabilization treatments are necessary before bottling to ensure sufficient conservation of wine quality and characteristics [6]. Apart from individual preferences, the quality and safety of wine are fundamental. Wine is a complex beverage composed of alcohol, sugars, acids, minerals, proteins, organic acids, and volatile and phenolic compounds, also called polyphenols, which play a crucial role in shaping the flavor profile of wine, contributing to its complexity, balance, and overall sensory experience [7].

Taking into consideration the safety of wine, the most common preservative is sulfur dioxide (SO_2_), which is widely used in various branches of the food industry, including fruit juices and fermentable beverages [8,9,10,11,12]. SO_2_ addition fulfills several functions, in which the most important are antioxidant and antimicrobial properties, as well as control of enzymatic reactions during wine production and storage [9,11,12]. However, the presence of SO_2_ in wine has raised concerns about inducing allergic symptoms and, thus, potential harmful effects in sensitive individuals. This has resulted in a general trend towards reducing or eliminating SO_2_, including widespread research efforts on alternative methods [5,8,10,11,13]. A great decrease in demand for preservatives in the final product may be enabled by the significant limitations of residual microbial charge and oxygen levels in the bottle [9]. Recent reports indicate various methods with potential in ensuring microorganisms’ stability and, therefore, possible application in order to decrease or replace the addition of sulfur dioxide to the wine. Generally, they may be divided into chemical additives, innovative physical techniques, and microbiological alternatives. One of the established technologies successfully used to eliminate microorganisms from food and beverages is ultraviolet irradiation [5,10,11].

UV-C rays (200–280 nm) are described as germicides due to their effective lethal effect on various microorganisms such as bacteria, protozoa, fungi, and algae, as well as viruses. The high-energy irradiation affects nucleic acids and proteins, causing their disruption. It also creates oxidative stress that is manifested by the creation of reactive oxygen species [14]. So far, UV-C has not only been considered as a prospective environmentally friendly and low-cost non-thermal technology for the decontamination of food products [15], but is also promising in increasing the shelf life of food and maintaining quality parameters while reducing the microbial load [16,17]. In recent years, it has been studied to remove undesirable microorganisms from wine as well [11]. Preliminary measurements confirm that matrix absorbance plays a crucial role in the inactivation of microorganisms by UV-C light. However, as excessive UV-C light intensities induce off-flavors in wines with high concentrations of riboflavin, it is necessary to optimize the UV-C doses delivered to the treated wines [5]. Certain authorities, including the European Food Safety Authority (EFSA), the US Food and Drug Administration (FDA), and the US Department of Agriculture, have already approved the use of UV radiation for microbial control in food products [17]. It is necessary to underline limitations, i.e., low penetration capacity and differentiation of transparency, that lead to problems with the establishment of the lowermost efficient UV dose and time of exposure. Only when optimal conditions are applied is it possible to maintain the physicochemical and sensory qualities of products [16]. Pilard et al. [10] highlighted an essential variability in UV-C response at inter- and intraspecific levels by testing 147 strains distributed among fourteen wine-related yeast species and six UV-C doses. An additional conclusion was that cellar-resident species, mainly associated with wine spoilage, showed higher sensitivity to UV-C exposure in comparison with vineyard-resident species. Higher UV-C doses increased the lag phase and decreased the growth rate and the maximal population [10].

Although UV is a promising technology for microbial inactivation, further improvements in overcoming limitations of the method, especially long treatment time or optical attenuation, are still required [18,19]. One studied approach focuses on multiple-hurdle technology, which combines various preservation techniques to achieve a synergistic effect that is greater than the impact of individual techniques. This approach ensures that microorganisms are unable to overcome the combined preservation methods [19,20,21,22,23]. The strategy has been found to be very efficient in meeting the production requirements of microbial safety, quality, and nutrition [22]. In line with hurdle technology, UV treatment is combined with additional preservation methods such as pulsed light, high hydrostatic pressure, mild heat [19], or sonication [21]. Another widely studied combination for microbial inactivation is photocatalysis, which is the process of UV light energy conversion into chemical energy under the presence of photocatalysts that accelerate photoreaction. A wide range of semiconductors have been explored for their efficiency as photocatalysts [19,24]. One that has been intensively studied and is considered the most active is titanium dioxide (TiO_2_) [19,24,25], which aroused great interest for its potential application in microbial inactivation/control of beverages, e.g., in drinking water and fruit juices [19,26]. From a mechanistic perspective, the use of photocatalysts is supposed to create oxidative stress on the cells through the generation of ROS. Additionally, nanomaterials may physically interact with cell envelopes [27]. However, the practical application of TiO_2_ photocatalysts carried the problem of them being lost and hard to re-collect from aqueous solution [28]. To resolve this problem, semiconductors were combined with conventional magnetic material, preserving the composite properties of pioneering precursors and enabling simple isolation after reaction through manipulation by an external magnetic field [25,28]. Various magnetic photocatalysts have been prepared as multi-layered composites, such as a core–shell material that contains a core (inner phase) coated by a shell (outer layer material), shell–core–shell, and multicores [25]. Fe_3_O_4_, with strong magnetization, had been proposed as a material suitable for the magnetic core [25,28]. Ferromagnetic materials are readily oxidized or undergo reduction during the catalytic reaction. However, several strategies have been proposed so far to overcome these obstacles [29]. The core–shell structure may be fabricated by introducing a protective layer of SiO_2_ between the Fe_3_O_4_ core and the TiO_2_ shell, which prevents electrons from being conveyed to the magnetic iron oxide and avoids iron leaching [29,30].

Our previous studies proposed photolysis or pure silica nanoparticles as potential solutions to ensure the microbiological safety of wine [31,32]. In the current research, we develop this idea further by testing the synergistic effect of these two methods for potential application before wine bottling.

The scientific objective of this work was to enhance yeast count reduction in wine by using ultraviolet light combined with TiO_2_-based magnetic photocatalysts. Therefore, we hypothesized that a synthetized Fe_3_O_4_/SiO_2_/TiO_2_ nanocomposite (NANO) will photocatalytically influence the viability of *Saccharomyces cerevisiae*, without altering wine chemistry. To validate the hypothesis, the experimental part included testing basic wine parameters, polyphenol content, and yeast neutralization. The individual effects of UV-C and the nanomaterial composite were examined to compare the impact of all factors on yeast viability, taking into account enhancements or declines in the neutralization process. The nanocomposite was synthesized by the immobilization of nanometric titanium dioxide on a mesoporous silica layer coated onto a magnetic nanoparticle core in order to readily extract it after the reaction. The application of photocatalysis supported by a nanomaterial composite with magnetic properties constitutes an innovative approach in the wine industry.

The practical objective of this research was to develop a technology that will effectively reduce the amount of yeast in wine and lead to a limitation of the sulfite dose, which may cause allergic reactions in some consumers. Elimination of yeast is an important procedure for both grape must and wine. As the essential step for the must, it enables the winemaker to use specific yeast strains in order to achieve the desired effects. However, yeast in wine is undesirable as it can contribute to the spoilage of bottled wine.

## 2. Results

### 2.1. Physical and Chemical Characterization of Iron Oxide–Silica/Titanium Oxide Core–Shell Nanocomposite

**Figure 1:**
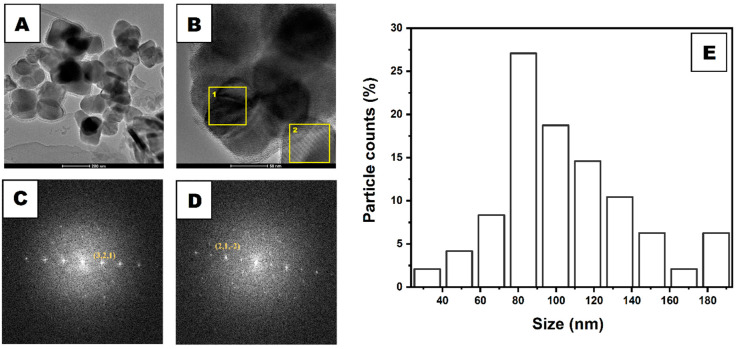
TEM (**A**) and HRTEM (**B**) images of iron oxide–silica/titanium oxide core–shell particles. FFT images (**C**,**D**) correspond to boxes 1 and 2 in image (**B**), respectively. Diagram (**E**) shows core–shell particle size distribution based on TEM analysis.

Detailed information on synthesized NANO was previously reported under the abbreviation Fe_3_O_4_/SiO_2_/TiO_2_ [33]. The TEM analysis of NANO revealed the morphology of the nanomaterial (Figure 1A). The round and square nanoparticles can be distinguished based on their shapes. The shell surrounding the nanoparticles can also be observed, which was assigned to the silica shell. The average particle size is 115 nm, and the size distribution of the nanoparticles (Figure 1E) reveals that most particles are in the range of 70–90 nm. High-resolution TEM images (HRTEM, Figure 1B) with corresponding fast Fourier transform (FFT) (Figure 1C,D) give insights into the crystallographic structure, providing a comprehensive understanding of the nanomaterial structure. Figure 1C,D corresponds to boxes (analyzed area) no. 1 and 2 from image 1B. The analysis reveals that the material comprises clear crystalline and amorphous particles. Based on d-spacing calculated from FFT, the presence of SiO_2_ (box no. 1 in Figure 1B) and Fe_2_O_3_ (box no. 2 in Figure 1B) was confirmed, according to ICDD no. 04-017-2316 and 01-084-2783, respectively. The XRD analysis proved that the structures were composed of iron oxide (maghemite—JCPDS no. 65-3107) and titanium dioxide (anatase—ICDD no. 03-065-5714; rutile—ICDD no. 03-065-1118). The zeta potential analysis showed that Fe_3_O_4_/SiO_2_/TiO_2_ particles had a negative charge at −19 mV. The surface charge of the particles was between pristine iron oxide (−1.1 mV) and iron oxide coated with silica (−37 mV) [33].

### 2.2. Effectiveness of Iron Oxide–Silica/Titanium Oxide Core–Shell Nanocomposite in Reduction in Saccharomyces cerevisiae Counts Under UV-C

**Figure 2:**
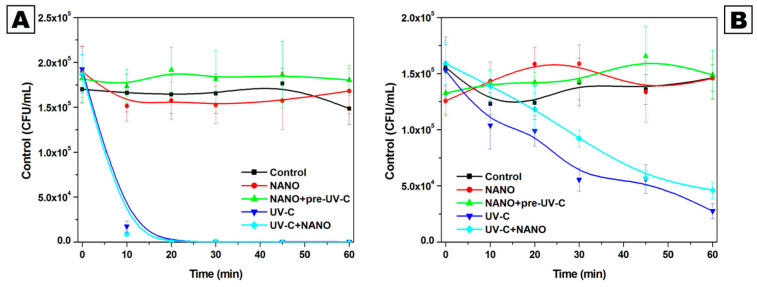
*S. cerevisiae* count in the samples of white (**A**) and red (**B**) wine after exposure to NANO, NANO+pre-UV-C, UV-C, and UV-C+NANO.

The influence of NANO, NANO+pre-UV-C, UV-C, and UV-C+NANO on the viability of *S. cerevisiae* in white and red wine was analyzed (Figure 2, Tables S1 and S2). The results revealed both UV-C and hurdle technology (UV-C+NANO) as the most sufficient and as the only distinct technologies in the reduction in yeasts compared to the control among the tested treatments.

In the case of white wine, the effect of the UV-C and UV-C+NANO methods resulted in a highly statistically significant (*p* < 0.0001) decrease in the count of yeasts ranging from 90% after 10 min of exposure up to 100% after the experiment. The samples exposed to NANO revealed a slight reduction in the amount of yeast 45 min into the experiment in comparison to the control. Then, after 45 min, the viability of yeasts exposed to NANO seemed to increase, while in the control this decreased, creating a gap between the samples of 13.2% at the 60th min of the study. Nevertheless, none of the observed differences were statistically significant. In turn, the yeast count under NANO+pre-UV-C maintained above the control throughout the experiment with statistically significant differences after 20 min (16.6%) and 60 min (21.3%) of exposure.

The general tendency of *S. cerevisiae* to respond to the tested methods in red wine was similar to that in white wine. UV-C and UV-C+NANO influenced the decrease in yeasts during the experiment, with a significant reduction in relation to the control after only 10 min (15.8%) and 30 min (35.1%), respectively. The final reduction achieved was 81% under UV-C and 68.3% under UV-C+NANO exposure. In turn, the amount of yeast treated with NANO increased significantly up to 20 min of the experiment and then stayed higher than or similar to the control with no statistical significance. The yeast number in red wine after NANO+preUV-C revealed a general tendency to increase throughout the experiment and stayed higher in relation to the control, with statistically significant results after 10 min (14.6%), 20 min (14.5%), and 45 min (21.9%) of exposure.

### 2.3. Microscopic Analysis of Interaction Between Yeasts Cells and Iron Oxide–Silica/Titanium Oxide Core–Shell Nanocomposite

**Figure 3:**
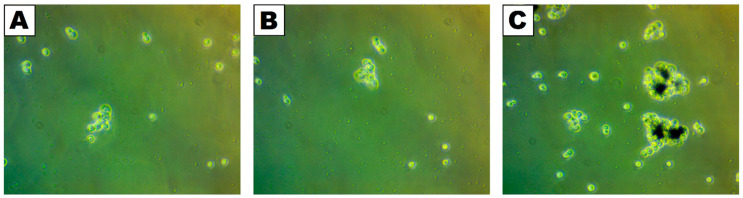
Phase-contrast images of *S. cerevisiae* cells in control sample (**A**), mixed with the nanomaterial and collected from the upper part (**B**), and mixed with the nanomaterial and collected from the bottom of the sample (**C**).

To analyze the interaction between yeast cells and the studied nanoparticles, phase-contrast images (Figure 3) were used. For the analysis, solution containing nanoparticles was divided into separate parts (solution near the surface—Figure 3B; from the bottom—Figure 3C). Additionally, *S. cerevisiae* cell solution was used as a control sample (Figure 3A). In the control and the sample exposed to NANO, as well as that collected close to the solution surface, no difference was noticed. On the other hand, in the sample collected from the bottom (Figure 3C), clear cell agglomerates around nanomaterials (black spots) could be noticed. The explanation for this is due to nanomaterials’ tendency to sediment over time. Thus, microscopic observations confirmed the increased cell agglomeration in the presence of the used nanomaterial.

### 2.4. General Parameters and Polyphenol Compounds in Wines After Treatment with UV-C and Hurdle Technology

UV-C and UV-C+NANO treatments had a statistically significant impact on alcohol content, volatile acidity, and fixed acidity in both white and red wines (Table 1). The alcohol content in wines revealed a tendency to decrease, especially after UV-C+NANO exposure. The volatile acidity and fixed acidity contents increased or decreased depending on the wine and treatment. Other parameters, such as glucose, fructose, pH, and malic acid, remained stable.

The white wine control sample contained approximately 84% less polyphenols than the red wine control sample, reflecting differences in the natural chemical composition of the two types of wine (Table 2). The polyphenol content in red wine samples also remained consistently higher after UV-C and UV-C+NANO treatment due to the presence of anthocyanins, which are the dominant group of polyphenols not present in white wine. The second most abundant group of detected compounds belonged to flavan-3-ols, which were present in red wine about twice as much as in white wine.

In white wine, flavan-3-ols showed significant changes in contents between the control, UV-C, and UV-C+NANO samples (Table 2). After applying UV-C, the flavan-3-ol content decreased by nearly 23%, which was caused by the loss of all examined compounds. Conversely, the application of UV-C+NANO influenced the growth of all compounds, resulting in a 57% increase in flavan-3-ol value, reaching a similar level to that found in red wine. In general, flavonols and stilbenes remained unaltered across all white wine samples, while the growth of phenolic acids after the application of both treatments was influenced only by an increase in caftaric acid (CA).

In the case of red wine, a significant decrease in anthocyanin content after applying UV-C and UV-C+NANO radiation was noted. After applying UV-C, the anthocyanin content dropped significantly by approximately 23%. Interestingly, the loss of anthocyanins under UV-C+NANO was relevantly less (15%) in relation to UV-C exposure, which was the result of a 55% increase in the content of malvidin 3-*O*-glucoside-5-*O*-glucoside (M3G5G) compared to the control value. In the same group, P3G showed a dramatic decrease after UV-C (58%) and UV-C+NANO (63%) exposure in relation to the control (Table 2).

A general declining tendency of the tested compounds under treatment was observed for flavan-3-ols, stilbenes, and phenolic acids. The application of both UV-C and UV-C+NANO methods in red wine relevantly decreased the content of flavan-3-ols by nearly 6% and 3%, respectively. A statistically meaningful loss of stilbenes was noted after UV-C+NANO due to a great decrease in *trans*- (tR) and *cis*-resveratrol (cR). Generally, the group of phenolic acids remained stable; however, looking at the compound level, a complete loss of coutaric acid (CoA) and caffeic acid (CaA) was observed after UV-C+NANO exposure.

In contrast, the flavonol content in red wine significantly increased in the sample exposed to UV-C+NANO by approximately 39% compared to the control and UV-C samples. All compounds were affected by photocatalysis with the exception of myricetin3-*O*-glucoside (MY3G), which remained stable.

## 3. Discussion

The tested nanomaterial being individually and initially exposed to UV-C light did not diminish the viability of *S. cerevisiae* despite lower counts observed in the samples. These drops could result from the agglomeration of cells with deposits of nanomaterial, which is visualized in Figure 2. Silica nanomaterial is known to have this effect on microorganisms. For example, agglomeration behavior was reported in the case of *Pseudomonas aeruginosa* contacted with silica/titania nanotubes [34]. Silica was previously shown to lose its stability in water samples, physiological salt, and bacteriological media, which may additionally promote the formation of agglomerates [35,36]. As suggested in another report, silica dissolving into smaller particles could be more accessible for internalization by microbial cells [35].

We assume that UV-C was mainly responsible for the reduction in yeast titers, as it was formerly suggested as an alternative to inactivate microorganisms in grape juices and wines [37]. Such irradiation was tested previously against *S. cerevisiae* in grape juice by Antonio-Gutiérrez et al. [38], who achieved a 5 log reduction in their system operating in continuous recirculation mode. A similar reduction in yeast in model wine under UV-C treatment was reported by Hirt et al. [39]. Our previous study revealed the potential of UV-C against *S. cerevisiae* in red wine; however, further optimization of the method’s efficiency was needed [31]. Recent reports indicate that combinations of nanomaterials with UV-C light have been commercially used to inactivate microorganisms in liquids. Chai et al. [40] successfully used TiO_2_ nanoparticles to remove yeasts, mold, and bacteria in freshly squeezed *Angelica keiskei* juice, though the combination had limited activity against sporulating bacteria (*Bacillus cereus*). Similar treatment was shown to be effective against *Listeria monocytogenes*, *Staphylococcus aureus*, *Escherichia coli*, *Salmonella* Typhimurium, and *Saccharomyces cerevisiae* in apple juice [41]. In our work, a combination of two methods, UV-C-assisted photocatalysis, was tested in order to improve the antimicrobial effect of UV-C light. The results showed that exposure to both UV-C and UV-C+NANO resulted in a statistically significant reduction in the amount of yeast. However, we did not observe an intensified effect of the combined technique in comparison to pure UV-C light. Furthermore, the photocatalysis revealed even less efficiency in the case of red wine. According to the above-mentioned results, the low efficiency of the photocatalyst in the hurdle technique may be explained by the agglomeration and internalization of nanoparticles by microorganisms [35].

In line with our results, Junqua et al. [5] observed UV-C irradiation as more efficient in microbiological inactivation in white and rosé wines than in red wine. Thereupon, the authors reported that the successful treatment of red wine required more than an eight-times-higher UV-C dose. Moreover, further results indicated that treatment did not alter the physicochemical and sensory properties of wines, including standard chemical parameters, color, the total polyphenol index (TPI), and anthocyanin content. It should also be emphasized that the UV-C treatments performed in the study lasted from 2 to 15 s [5]. The lower efficiency of UV-C light penetration in red wines is related to anthocyanins. In turn, Ramesh et al. [42] found the lowest loss of health-related compounds (vitamin C, total phenolic content, total antioxidant capacity) in white grape juice under 60 min of UV-A treatment, followed by 40 min of UV-A-assisted photocatalysis and 20 min of UV-C. The differences between exposure time resulted from the necessity of a comparison of juice quality under the same level of target microorganism reduction. Moreover, the authors observed no changes in pH and titratable acidity and an increase in total soluble solids under all UV treatments. In our study, statistical changes were observed in alcohol content, volatile acidity, and fixed acidity, while glucose, fructose, pH, and malic acid remained stable under UV-C-induced treatments. A noteworthy point is the reduction in alcohol content. This suggests a potential application of the examined methods in the dealcoholization of wine, another challenge in the wine industry to meet limitations for a specific wine style and consumers’ demand for reduced-alcohol or non-alcoholic wines with good sensory qualities [43,44,45]. Currently, there are no published scientific studies in reputable journals that analyze the effect of UV-C treatment or UV-C combined with nanotechnology on the reduction in alcohol content in wine. Available studies primarily focus on the impact of UV-C radiation on the microbiological and chemical properties of wine, such as the reduction in microorganisms or changes in phenolic and aromatic composition, but they do not directly address the reduction in alcohol levels. Therefore, additional research is necessary to understand the mechanisms by which UV-C and UV-C+NANO treatments could influence the alcohol content in wine, as well as to assess the potential benefits and limitations associated with such methods. Additionally, in contradiction to the majority of previous research that provided insights only into general polyphenol content, our work has shown that wine chemistry may be altered by UV-C treatment, including polyphenol concentrations and the fractions of polyphenol types in the groups. Pala and Toklucu [46] reached a complete inactivation of microbial load in grape juices, both white and red, under the same dose of UV-C treatment and with no significant changes in antioxidant capacity, total phenolics, titratable acidity, soluble solids, and pH. The authors also weighed UV-C irradiation against heat treatment and observed losses in monomeric anthocyanins of red grape juice by 8.7% and 11.8%, respectively. As reported by Diesler et al. [47], anthocyanins degrade under UV-C radiation, leading to a loss of color intensity and a negative impact on wine’s sensory attributes. Thereby, UV-C can break down anthocyanins into simpler compounds [48,49]. Our results showed a decrease in the content of total anthocyanins by 23% and 15% after treatment with UV-C and UV-C+NANO, respectively. The loss of these compounds was 8% lower in the sample after UV-C+NANO. A similar observation was made on flavan-3-ols. This group of compounds is responsible for stabilizing the color of red wine as they can interact with anthocyanins to form stable pigments that enhance its color. Therefore, the decomposition of flavan-3-ols may diminish color stability, contributing to decreases in anthocyanin content and a consequent fading of wine color. Our results may suggest that a general reduction in anthocyanins followed on the decomposition of flavan-3-ols was triggered by photochemical reactions under UV-C. This effect was reduced by the addition of nanomaterials, which affected the turbidity of the wine. Thereupon, a consistent response of these two groups of compounds to the tested methods seems to be logical. In contrast, our previous work showed that each of the examined anthocyanin compounds decreased after UV-C irradiation [31]. Additionally, as the profile of the examined flavan-3-ols was formerly limited in comparison to our current study, we observed a significant increase in total flavan-3-ols in red wine that resulted from the growth of (−)-epicatechin. Similarly, with this research, a significant increase in the contents of this specific compound and group was found in white wine after photocatalysis.

A particularly noteworthy observation at the compound level of our results was the specific increase in malvidin 3-*O*-glucoside-5-*O*-glucoside content after treatments, especially after UV-C+NANO. This result suggests that the nanocomposite may facilitate the release of this compound from other phenolic complexes during the photocatalytic process. A similar phenomenon has been proposed in previous research, where nanomaterials were shown to interact with polyphenols and enhance their stability under stress conditions [50].

However, the synthesis and degradation mechanisms of polyphenols in wine are not well studied so far; hence, it should be considered that they might also be similar to those observed in plants. Looking through the prism of well-known anthocyanin biosynthesis mechanisms in plants, these groups of compounds tend to accumulate in the sun-exposed side of fruits to protect them from photoinhibition and photobleaching under light stress [51,52,53,54,55]. Different light intensity levels and spectra, such as UV-A, blue, or red light, were reported to increase the anthocyanin content of fruits and vegetables [56]. In line with this mechanism, behind the increase in malvidin might be its response to stress factors. In contrast, the significant decrease in peonidin 3-*O*-glucoside content under both UV-C and UV-C+NANO treatment highlights the different sensitivity of various anthocyanins to UV radiation and nanocomposite treatments. Similar findings have been reported by Modesti et al. [57], who noted that specific anthocyanins are more susceptible to degradation under light exposure.

Guerrero et al. [58] indicated that even the UV-C treatment of grapes may change resveratrol content, and this technique could be used to produce stilbene-enriched grapes. In contradiction, our study revealed no impact of UV-C on the content of stilbenes in wines. However, the application of UV-C+NANO resulted in decreases in resveratrol contents, which was significant in red wine.

## 4. Materials and Methods

### 4.1. Experimental Design

**Figure 4:**
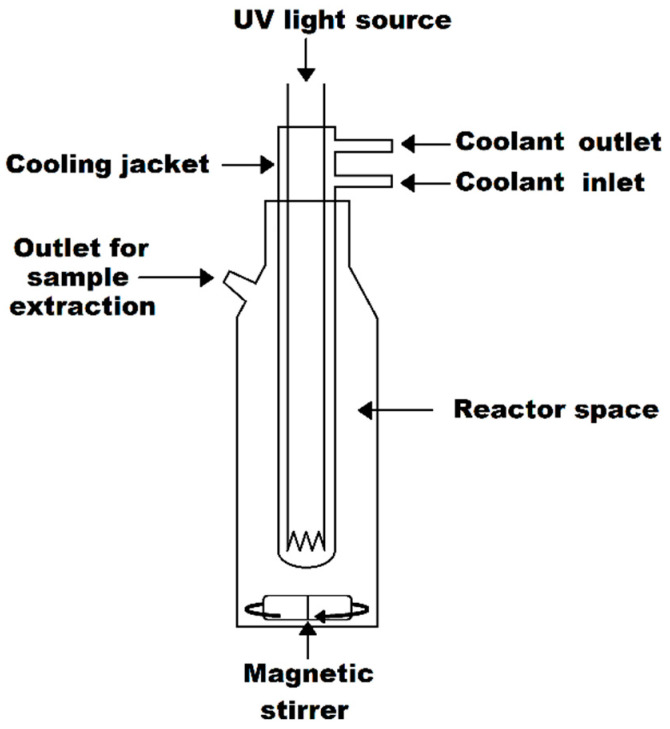
The scheme of used UV-C inner-irradiation-type reactor.

The experiment included Muscaris and Cabernet Cortis wines, white and red types, respectively, purchased from Kojder Vineyards, Bielice, Poland. The Muscaris and Cabernet Cortis wines were not sulfurized and contained 12 and 11 mg/L of free SO_2_ as well as 24 and 10 mg/L of total SO_2_, respectively. In the winery, the wine undergoes natural sedimentation by lowering the temperature and racking the wine from the lees into stacked fermentation tanks using a gravitational method. Directly before the experiment, wine was filtered through a PES syringe filter with a pore size of 0.22 µm (qpore, Bionovo, Legnica, Poland) and then inoculated with commercial yeast, *S. cerevisiae* EnartisFerm SC (Enartis, San Martino NO, Italy). The suspension from the 48 h culture of *S. cerevisiae* was added to wine samples (500 mL) to reach a final cell density of approximately 1.6 × 10^5^ CFU/mL. The efficiency of the hurdle technique (UV-C combined with photocatalyst) in microbial deactivation was referred to individual methods and control. Thereby, five experimental setups were performed, each analyzed separately for white and red wine: (1) wine (control); (2) wine + Fe_3_O_4_/SiO_2_/TiO_2_ (NANO); (3) wine + NANO after an hour of initial exposure to UV-C (NANO+pre-UV-C); (4) wine + UV-C photolysis (UV-C); (5) wine + UV-C assisted with NANO photocatalysis (UV-C+NANO). The counts of *S. cerevisiae* in all samples were examined from samples collected during an hour-long exposure to the experimental factor at selected time points (0, 10, 20, 30, 45, and 60 min). The photoactive ability was determined under UV-C light, carried out in an inner-irradiation-type reactor (Figure 4) with a capacity of 500 mL, equipped with a low-pressure 15 W mercury lamp of wavelength 254 nm, delivering energy in the range of 58–78 mJ/cm^2^ [31]. A cooling jacket was used to avoid sample overheating, and thereupon, the temperature of the stirred suspensions was maintained under 20 °C. Nanomaterials were sonicated for an hour in a one-tenth volume of sample each time before the experiment started and then added to the wine inoculated with yeasts. Similarly, after hour-long irradiation, the nanocomposite initially treated with UV-C light was immediately added to the wine. The nanomaterial was studied in the final concentrations of 150 mg/L. Polyphenols and basic oenological parameters were studied in controls and samples processed with UV-C.

### 4.2. Reagents and Standards

Titanium (IV) butoxide (TBT), hexadecyl (trimethyl)azanium bromide (CTAB), and iron oxide were purchased from Sigma Aldrich (MERCK, Darmstadt, Germany). The iron oxide particle size distribution was in the range of 50–100 nm (purity 97%, according to information provided by the supplier). Ethanol, propanol, and 30–32% ammonia solution were provided by Chempur (Piekary Śląskie, Poland).

### 4.3. Synthesis and Analyses of Iron Oxide–Silica/Titanium Oxide Core–Shell Nanocomposite

The synthesis of core–shell, iron oxide–silica/titanium oxide (NANO) particles was reported previously [33]. As reported, 2 g of Fe_3_O_4_ and 0.930 g of hexadecyl (trimethyl)azanium bromide (CTAB) were dispersed in 200 mL of ethanol using a stirrer and ultrasound. Further, the obtained suspension was mixed with 300 mL of water and 2.5 mL of ammonia solution (NH_3_ × H_2_O). Simultaneous stirring and sonication were used to retain the dispersion of iron oxide particles in the ethanol–water solution. Further, 1.5 mL of tetraethyl orthosilicate was added and kept stirring for 18 h at room temperature. After stirring, the obtained particles were separated with a magnet and air-dried. The dried material was annealed at 500 °C for 2 h to burn out CTAB blocking the pores.

In the next steps, empty pores of the mesoporous silica shell (Fe_3_O_4_-SiO_2_) were functionalized with titanium dioxide according to the previously described method [35,59]. According to the method, Fe_3_O_4_-SiO_2_ structures were added to the concentrated TBT and sonicated for 2 h in an ultrasonic water bath at 50 °C. After sonication, Fe_3_O_4_-SiO_2_ structures were separated from the concentrated TBT by a magnet and washed with isopropanol. Afterward, nanostructures were washed with ethanol to hydrolyze the remaining TBT and dried in air at 60 °C. The dried material was heated in an inert atmosphere for 2 h at 400 °C.

The morphology of the nanomaterial composite was examined with a transmission electron microscope (TEM; Tecnai G2 F20 S-TWIN, FEI, Hillsboro, OR, USA), detailed described in our previous articles [32,33,36]. The crystal structure of the samples was characterized by an X-ray powder diffractometer (XRD, Aeris, Malvern Panalytica, Malvern, UK), and zeta potential was determined by a Zetasizer Nano ZS (Malvern Instruments, Malvern, UK) [33].

### 4.4. Preparation of Saccharomyces cerevisiae Cultures

The *Saccharomyces cerevisiae* strain isolated from EnartisFerm SC (Enartis, San Martino NO, Italy) was used as a model microorganism. The choice of this yeast was practical because it is a key microorganism in wine fermentation that is also cultivable and quantitatively measurable. This *S. cerevisiae* strain was isolated on Sabouraud Dextrose Agar (Emapol, Gdańsk, Poland) supplemented with chloramphenicol (0.05 g/L) and cycloheximide (0.1 g/L) and stored in Sabouraud Dextrose Broth (Emapol, Gdańsk, Poland) with 20% (*v*/*v*) glycerol at −20 °C.

For the experiment, *S. cerevisiae* was revived by streaking onto Sabouraud Dextrose Agar (Emapol, Gdańsk, Poland) supplemented with chloramphenicol (0.05 g/L) and cycloheximide (0.1 g/L) and cultured at 25 °C for 48 h. Then, yeast suspension with a turbidity of 3 McFarland standard (approx. 3 × 10^8^ CFU/mL) was prepared in 0.85% (*w*/*v*) NaCl solution.

### 4.5. Post-Treatment Analyses of Samples

#### 4.5.1. *Saccharomyces cerevisiae* Counts

To determine the number of viable yeasts, decimal dilutions in 0.85% (*w*/*v*) NaCl solution were made immediately after sample collection and plated on Sabouraud Dextrose Agar (SDA, Emapol, Gdańsk, Poland) supplemented with chloramphenicol (0.05 g/L) and cycloheximide (0.1 g/L). The cultures were incubated at 25 °C for 48 h, and the grown colonies were counted.

Aggregation of cells was observed under a phase-contrast microscope (Delta Optical L1000, Mińsk Mazowiecki, Poland). For these observations, Fe_3_O_4_/SiO_2_/TiO_2_ was co-incubated with cells for 60 min. Afterwards, samples of 5 µL were collected, spotted on microscope slides, and covered with a coverslip. At least three representative images for each case were captured and saved. Specimens were observed under ×1000 magnification.

#### 4.5.2. Basic Oenological Parameter Determination

Alcohol strength by volume (OIV-MA-AS312-01), glucose and fructose (OIV-MA-AS311-02), total acidity (OIV-MA-AS313-01), L-malic acid (OIV-MA-AS313-11), volatile acidity (OIV-MA-AS313-02), and pH (OIV-MA-AS313-15) were determined according to the official OIV methods [60].

#### 4.5.3. Polyphenol Determination with the UPLC-PDA-MS/MS Method

The applied liquid chromatography technique was combined with a PDA detector and TQD-ESI-MS/MS [61]. The separation was carried out using a BEH C18 column (100 mm × 2.1 mm i.d., 1.7 µm, Waters) kept at 50 °C. For the anthocyanin investigation, the following solvent system was applied: mobile phase A (2% formic acid in water *v*/*v*) and mobile phase B (2% formic acid in 40% ACN in water *v*/*v*). For other polyphenolic compounds, a lower concentration of formic acid was used (0.1% *v*/*v*). The gradient program was set as follows: 0 min 5% B, from 0 to 8 min linear to 100% B, and from 8 to 9.5 min for washing and back to initial conditions. The injection volume of the samples was 5 µL (partial loop with needle overfill) and the flow rate was 0.35 mL/min. The following parameters were used for TQD: capillary voltage: 3.5 kV; con voltage: 30 V in positive and negative mode; the source was kept at 250 °C and the desolvation temperature was 350 °C; con gas flow: 100 L/h; and desolvation gas flow: 800 L/h. Argon was used as the collision gas at a flow rate of 0.3 mL/min. The characteristic UV spectra were collected at the following wavelengths: λ = 520 nm, anthocyanins; λ = 320, phenolic acids; λ = 360, flavonols; and λ = 280, flavan-3-ols. Quantification was achieved by injection of solutions of known concentrations ranging from 0.05 to 5 mg/mL (R2  ≤  0.9998) of phenolic compounds as standards. Waters MassLynx software v.4.1 was used for data acquisition and processing. In turn, in the white wine, a total of 31 polyphenols were detected due to the presence of additional procyanidin trimers (*m*/*z* = 865) and the lack of anthocyanins in the qualitative composition. Flavan-3-ols were the most numerous group of compounds, which included monomers such as (+)-catechine and (−)-epicatechine (*m*/*z* = 285) [62] and procyanidins such as A1 and A2 (*m*/*z* = 577) [63], B1 and B2 (*m*/*z* = 577) [64], and C1 and C2 (*m*/*z* = 577) [65], as well as procyanidin trimers (4 compounds; *m*/*z* = 865) [64]. The next class of compounds was monoglucosides and diglucosides of flavonols, which were illustrated by the presence of myricetin (*m*/*z* = 317), quercetin (*m*/*z* = 301, and *m*/*z* = 303), and isorhamnetin (*m*/*z* = 315) derivatives with the characteristic loss of rhamnoside (146 Da), pentoside (132 Da), hexoside (162 Da), and a glucuronic acid moiety (176 Da) [62]. Monoglycosides and diglycosides of anthocyanins present in red wine gave fragmentation ions characteristic of the following substances: cyanidin (*m*/*z* = 287), delphinidin (*m*/*z* = 303), peonidin (*m*/*z* = 301), and malvidin (*m*/*z* = 331). These relationships were confirmed by the characteristic loss of a hexoside moiety (162 Da) and previously reported by Błaszak et al. [62] and Kapusta et al. [64]. Among the detected phenolic acids, 2 compounds belonged to hydroxybenzoic acid derivatives as gallic acid (*m*/*z* = 125) and protocatechuic acid (*m*/*z* = 109), and 5 compounds belonged to hydroxycinnamic acid derivatives as *p*-coumaric (*m*/*z* = 119) and caffeic acids (*m*/*z* = 109), coutaric acid (*m*/*z* = 163), and caftaric acid (*m*/*z* = 179). These relationships are characteristic of red and white wines and were previously confirmed by Błaszak et al. [62] and Kapusta et al. [64]. The last group was stilbenes, which indicated 4 compounds—*cis* and *trans* piceid (*m*/*z* = 389) and *cis* and *trans*-resveratrol (*m*/*z* = 227) [62,64].

The following 36 compounds were identified: Cya3-*O*-glu-5-*O*-glu—C3G5G; Delpinidin3-*O-*glucoside—D3G; Peonidin3-*O*-glucoside-5-*O*-glucoside—P3G5G; Malvidin3-*O*-glucoside-5-*O*-glucoside—M3G5G; Peonidin3-*O*-glucoside—P3G; Malvidin3-*O*-glucoside—M3G; Myricetin3-*O*-rutinoside—MY3R; Myricetin3-*O*-glucoside—MY3G; Quercetin3-*O*-glucuronide—Q3GQ; Isorhamnetin3-*O*-glucoside—I3G; Quercetin3-*O*-glucoside—Q3G; Quercetin3-*O*-rutinoside—Q3R; Dihydroquercein3-*O*-rhamnoside—DQ3RH; ProcyanidinB1—PB1; ProcyanidinA1—PA1; Procyanidin trimer Ptr1; (+)-catechin—(+)C; Procyanidin trimer Ptr2; Procyanidin B2—PB2; Procyanidin A2—PA2; (−)-epicatechin—(−)e; Procyanidin trimer Ptr3; Procyanidin trimer Ptr4; Procyanidin C1—PC1; Procyanidin C2—PC2; *trans*-piceid—tPi; *cis*-piceid—cPi; *trans*-resveratrol—tR; *cis*-resveratrol—cR; gallic acid—GA; protocatechuic acid—PcA; caftaric acid—CA; coutaric acid—CoA; caffeic acid—CaA; *p*-coumaric acid—*p*CuA; coumaric acid—CuA.

## 5. Statistics

Statistical descriptions included means and standard deviations that were calculated for all numerical data. The minimum number of experimental repetitions was n = 3 unless stated otherwise in the manuscript. Data are presented in this manuscript as mean values ± standard error of the mean (SEM). Statistical differences between data were determined using a two-way analysis of variance (ANOVA) using GraphPad Prism 9.0 (GraphPad Software Inc., Boston, MA, USA). Tukey’s HSD (Honestly Significant Difference) post hoc test was used for multiple data comparisons. All differences were considered statistically significant when the *p*-value was less than 0.05.

## 6. Conclusions

Comparing the effects of nanomaterials, UV-C-assisted photolysis, and hurdle technology on the viability of *S. cerevisiae* in white and red wine, it can be concluded that the fastest and most effective method of eliminating yeast from both types of wine is exposure to UV-C. The UV-C+NANO method is as effective as UV-C but requires longer exposure of yeast to irradiation in red wines.

Our findings suggest that while UV-C radiation is effective for microbial control, it may also lead to the loss of important phenolic compounds, reducing the wine’s antioxidant properties and sensory complexity.

In turn, detected differences in the chemical composition of wines after applying the hurdle technology, joining UV-C irradiation with core–shell iron oxide–silica/titanium oxide nanoparticles, revealed a potential application in controlling the polyphenol composition and alcohol reduction. Thereupon, when further examined, the method may serve a purpose in wine preservation or dealcoholization.

Th significance of wine’s initial chemical composition should be emphasized, including fractions of groups as well as individual compounds, in consecutive biosynthetic pathways and degradation mechanisms. Additionally, omnidirectional changes have to be considered under different stress factors and interplays between compounds. Further research is needed to fully understand how nanocomposites interact with phenolic compounds and optimize their use in wine processing to maintain or enhance wine quality.

## Figures and Tables

**Table 1:** General parameters of white and red wine before and after UV-C and UV-C+NANO treatment.

Parameters	White Wine			Red Wine		
	Control	UV-C	UV-C+NANO	Control	UV-C	UV-C+NANO
Alcohol content	12.13 b *	12.13 b	12.00 a	13.13 b	12.60 a	12.63 a
Glucose + Fructose	0.027 a	0.030 a	0.027 a	0.027 a	0.030 a	0.027 a
Volatile acidity	0.50 a	0.51 b	0.52 b	0.51 a	0.53 b	0.50 a
Fixed acidity	5.05 a	5.04 ab	5.08 b	4.50 b	4.44 a	4.43 a
pH	3.42 a	3.42 a	3.42 a	3.69 a	3.70 a	3.71 a
*L*-Malic acid	0.100 a	0.097 a	0.110 a	0.040 a	0.040 a	0.037 a

* Mean values denoted by the same letter do not differ statistically significantly at 0.05 according to Tukey’s test.

**Table 2:** Concentrations of polyphenols in white and red wine before and after UV-C and UV-C+NANO treatment.

Parameters [mg/L]	White Wine			Red Wine		
	Control	UV-C	UV-C+NANO	Control	UV-C	UV-C+NANO
Anthocyanins	**-**	**-**	**-**	197.4 c *	152.2 a	167.1 b
C3G5G **	**-**	**-**	**-**	4.37 a	5.68 b	6.41 b
D3G	**-**	**-**	**-**	16.93 b	10.69 a	11.15 a
P3G5G	**-**	**-**	**-**	15.09 a	17.05 a	16.36 a
M3G5G	**-**	**-**	**-**	36.62 a	48.15 b	56.81 c
P3G	**-**	**-**	**-**	77.68 b	32.79 a	28.89 a
M3G	**-**	**-**	**-**	46.75 ab	37.35 a	47.99 b
Flavonols	1.34 a	1.35 a	1.53 a	2.05 a	2.05 a	2.84 b
MY3R	0.43 a	0.42 a	0.49 a	0.80 a	0.85 a	1.18 b
MY3G	0.06 a	0.08 b	0.06 a	0.10 a	0.10 a	0.10 a
Q3GQ	0.015 b	0.010 b	0.000 a	0.010 a	0.010 a	0.055 b
I3G	0.76 a	0.76 a	0.89 b	1.05 a	0.99 a	1.38 b
Q3G	0.065 a	0.065 a	0.075 a	0.095 a	0.105 a	0.120 b
Q3R	0.01 a	0.01 a	0.01 a	**-**	**-**	**-**
DQ3RH	-	-	0.01	**-**	**-**	0.01
Flavan-3-ols	36.77 b	28.47 a	57.60 c	65.30 b	59.80 a	61.52 a
PB1	10.36 b	8.40 a	11.86 b	14.71 b	13.03 a	14.16 ab
PA1	8.58 b	7.44 a	10.78 c	13.87 a	14.92 a	12.95 a
Ptr1	0.42 b	0.29 a	0.89 c	1.29 b	0.92 a	1.17 ab
(+)-C	0.97 b	0.51 a	2.26 c	2.62 a	2.49 a	2.49 a
Ptr2	0.22 b	0.13 a	0.63 c	0.88 b	0.74 a	0.84 ab
PB2	1.11 b	0.63 a	3.22 c	4.67 b	3.50 a	4.11 b
PA2	3.85 b	2.63 a	8.61 c	10.03 b	8.63 a	9.80 b
(-)-E	3.20 b	2.32 a	6.60 c	7.49 a	7.35 a	7.10 a
Ptr3	2.25 b	1.61 a	3.62 c	-	-	-
Ptr4	1.76 b	0.85 a	3.84 c	4.39 b	3.51 a	4.05 b
PC1	3.56 ab	3.40 a	4.02 b	3.89 b	3.33 a	3.49 a
PC2	0.52 b	0.28 a	1.29 c	1.48 a	1.39 a	1.39 a
Stilbenes	1.87 a	1.92 a	1.87 a	3.43 b	3.33 b	2.92 a
tPi	0.17 a	0.16 a	0.16 a	0.22 a	0.22 a	0.29 b
cPi	1.01 a	1.02 a	1.10 b	1.54 a	1.52 a	2.06 b
tR	0.13 a	0.14 a	0.12 a	0.26 b	0.30 c	0.06 a
cR	0.57 b	0.62 b	0.50 a	1.42 b	1.31 b	0.51 a
Phenolic acids	7.76 a	8.39 b	8.29 b	10.00 a	9.11 a	8.86 a
GA	4.61 a	5.05 a	4.99 a	6.65 ab	5.95 a	7.72 b
PcA	0.11 a	0.09 a	0.09 a	0.15 a	0.19 a	0.15 a
CA	2.25 a	2.47 b	2.46 b	2.41 b	2.21 b	0.01 a
CoA	0.66 a	0.65 a	0.62 a	0.62 a	0.59 a	-
CaA	0.11 a	0.10 a	0.11 a	0.12 a	0.12 a	0.72 b
*p*CuA	0.01 a	0.01 a	0.01 a	0.02 a	0.02 a	0.14 b
CuA	0.03 a	0.03 a	0.03 a	0.04 a	0.04 a	0.12 b
Total	44.74 a	40.11 a	69.28 b	278.2 c	226.4 a	243.2 b

* See Table 1. ** Abbreviations and full names of all compounds are presented in the Materials and Methods section.

## Data Availability

All data supporting the findings of this study are available within the paper and in Supplementary Materials.

## Supplementary Materials

The following supporting information can be downloaded at https://www.mdpi.com/article/10.3390/molecules30020221/s1: Table S1: *S. cerevisiae* count in white wine after exposure to NANO, NANO+pre-UV-C, UV-C and UV-C+NANO; Table S2: *S. cerevisiae* count in red wine after exposure to NANO, NANO+pre-UV-C, UV-C and UV-C+NANO.
