# Supplementary material for: UV-C and Nanomaterial-Based Approaches for Sulfite-Free Wine Preservation: Effects on Polyphenol Profile and Microbiological Quality
Source: Molecules. 2025 Jan 8;30(2):221. doi: 10.3390/molecules30020221 (PMC11767371; doi:10.3390/molecules30020221)
Supplement: Supplementary file 1 [file molecules-30-00221-s001.zip › Table S1.pdf]

Table S1. *S. cerevisiae* count in white wine after exposure to NANO, NANO+pre-UV-C, UV-C and UV-C+NANO.

Time	Control	NANO		NANO+pre-UV-C		UV-C		UV-C+NANO	
	AA	AA	%	AA	%	AA	%	AA	%
0 min	1.7×10^5	1.9×10^5		1.8×10^5		1.9×10^5		1.9×10^5	
10 min	1.7×10^5	1.5×10^5	8.7	1.7×10^5	4.4	1.7×10^4 ****	89.5	9.2×10^3 ****	94.4
20 min	1.6×10^5	1.6×10^5	4.1	1.9×10^5 **	16.6	2×10^2 ****	99.9	3×10^2 ****	99.8
30 min	1.7×10^5	1.5×10^5	7.8	1.8×10^5	9.8	2×10^2 ****	99.9	6×10^2 ****	99.6
45 min	1.8×10^5	1.6×10^5	10.9	1.9×10^5	5.7	4×10^2 ****	99.8	2×10^2 ****	99.9
60 min	1.5×10^5	1.7×10^5	13.2	1.8×10^5 **	21.3	2×10^2 ****	99.9	0 ****	100

(green) decrease

(red) increase

AA - arithmetic average; % - compared to the control

P value: * $p < 0.05$, ** $p < 0.01$, *** $p < 0.001$, **** $p < 0.0001$, one-way ANOVA followed by the Tukey's multiple-comparison test
