# Supplementary material for: UV-C and Nanomaterial-Based Approaches for Sulfite-Free Wine Preservation: Effects on Polyphenol Profile and Microbiological Quality
Source: Molecules. 2025 Jan 8;30(2):221. doi: 10.3390/molecules30020221 (PMC11767371; doi:10.3390/molecules30020221)
Supplement: Supplementary file 1 [file molecules-30-00221-s001.zip › Table S2.pdf]

Table S2. *S. cerevisiae* count in red wine after exposure to NANO, NANO+pre-UV-C, UV-C and UV-C+NANO.

Time	Control	NANO		NANO+pre-UV-C		UV-C		UV-C+NANO	
	AA	AA	%	AA	%	AA	%	AA	%
0 min	1.5×10^5	1.3×10^5		1.3×10^5		1.5×10^5		1.6×10^5	
10 min	1.2×10^5	$1.4 \times 10^{5*}$	16.4	$1.4 \times 10^{5*}$	14.6	$1.0 \times 10^{5*}$	15.8	1.4×10^5	12.8
20 min	1.2×10^5	$1.6 \times 10^{5***}$	28	$1.4 \times 10^{5*}$	14.5	$9.9 \times 10^{4***}$	20.2	1.2×10^5	4.5
30 min	1.4×10^5	1.6×10^5	11.6	1.4×10^5	0.5	$5.6 \times 10^{4****}$	60.9	$9.2 \times 10^{4****}$	35.1
45 min	1.4×10^5	1.3×10^5	1.7	$1.7 \times 10^{5**}$	21.9	$5.6 \times 10^{4****}$	58.7	$5.7 \times 10^{4****}$	57.8
60 min	1.5×10^5	1.5×10^5	0.2	1.5×10^5	1.9	$2.8 \times 10^{4****}$	81	$4.6 \times 10^{4****}$	68.3

(green) decrease

(red) increase

AA - arithmetic average; % - compared to the control

P value: * $p < 0.05$, ** $p < 0.01$, *** $p < 0.001$, **** $p < 0.0001$, one-way ANOVA followed by the Tukey's multiple-comparison test
